# Complete Genome Sequence of Zika Virus Isolated from Semen

**DOI:** 10.1128/genomeA.01116-16

**Published:** 2016-10-13

**Authors:** Barry Atkinson, Victoria Graham, Rory W. Miles, Kuiama Lewandowski, Stuart D. Dowall, Steven T. Pullan, Roger Hewson

**Affiliations:** a National Infection Service, Public Health England, Porton Down, Wiltshire, United Kingdom; b National Institute for Health Research, Health Protection Research Unit in Emerging and Zoonotic Infections, Liverpool, United Kingdom

## Abstract

Zika virus (ZIKV) is an emerging pathogenic flavivirus currently circulating in numerous countries in South America, the Caribbean, and the Western Pacific Region. Using an unbiased metagenomic sequencing approach, we report here the first complete genome sequence of ZIKV isolated from a clinical semen sample.

## GENOME ANNOUNCEMENT

Since October 2013, an outbreak of Zika virus (ZIKV) has affected 68 countries across South America, the Caribbean, and the Western Pacific Region (1). Nearly half a million confirmed and suspected cases have been reported; while transmission to humans typically occurs via the bite of an infected mosquito, 11 countries have reported cases of non-mosquito-borne transmission (1, 2). ZIKV RNA has been identified in semen from infected males, including detection at more than two months post onset of symptoms (3–5). However, to date, no ZIKV sequence greater than 300 bases long from semen has been made publically available.

A diagnostic semen sample provided 13 days post onset of symptoms from a patient returning from Guadeloupe was identified as positive for ZIKV RNA with a high copy number. ZIKV was isolated from the clinical semen sample in the C6/36 cell line. Isolation was not successful directly in the Vero cell line; however, infected C6/36 supernatant did propagate in Vero cells and produced cytopathic effect. Supernatant was removed for metagenomic analysis on days 5 and 7 in C6/36 cells and on day 3 in Vero cells. Eighty microliters of supernatant was inactivated in 320 µl of AVL buffer (Qiagen) and purified using the EZ1 virus minikit version 2.0 (Qiagen).

RNA extracts were DNase digested and purified as described previously (6). cDNA was prepared from total RNA using the method of Greninger et al. (7), with the amendment that an alternative polymerase was used in round B (35 µl of water, 5 µl of 10× reaction mixture, 1 µl of 100 µM primer B, 1 µl of dimethyl sulfoxide [DMSO], 2.5 µl of 12.5 mM dinucleoside triphosphate [dNTP], 0.5 µl of Sigma Aldrich AccuTaq LA DNA polymerase, and 5 µl of round A-labelled cDNA). The reaction conditions were 98°C for 30 s; 30 cycles of 94°C for 15 s, 50°C for 20 s, and 68°C for 5 min; and 68°C for 10 min. cDNA (1.5 ng) was prepared for sequencing, according to the Illumina Nextera XT protocol, and the 2 × 150-bp paired-end library run on an MiSeq (Illumina).

Reads were quality trimmed to a minimum score of Q30 across the read. BWA version 0.7.5 (8) was used to map 913,794 reads from the day 3 Vero sample to the ZIKV PF13/251013-18 genome (GenBank accession no. KX369547), of which 2.4% mapped to the reference, giving 99.3% genome coverage at a minimum depth of 5×, from which a consensus genome sequence was produced. All analyses were performed using a local instance of the Galaxy Project (9–11). Significant genome coverage was also achieved for C6/36 samples, although only 77% of bases had a depth of five reads or greater; all callable bases were homologous to Vero data.

Sequence data for the virus isolated from a clinical semen sample align well with other sequences of ZIKV from the outbreak, including those from the Caribbean region, where the patient acquired the infection. The characterization of ZIKV isolated from semen samples will help improve our understanding of possible viral polymorphisms resulting from infection in different cellular environments. The isolated virus will be available imminently from the National Collection of Pathogenic Viruses (12) and the European Virus Archive (13).

### Accession number(s).

The complete genomic sequence has been deposited in GenBank under accession no. KX673530.
